# Added value of 3D-vision during robotic pancreatoduodenectomy anastomoses in biotissue (LAEBOT 3D2D): a randomized controlled cross-over trial

**DOI:** 10.1007/s00464-020-07732-z

**Published:** 2020-07-13

**Authors:** Maurice J. W. Zwart, Leia R. Jones, Alberto Balduzzi, Kosei Takagi, Aude Vanlander, Peter B. van den Boezem, Freek Daams, Camiel Rosman, Daan J. Lips, Arthur J. Moser, Melissa E. Hogg, Olivier R. C. Busch, Martijn W. J. Stommel, Marc G. Besselink, For the Dutch Pancreatic Cancer Group

**Affiliations:** 1 Department of Surgery, Cancer Center Amsterdam, Amsterdam UMC, University of Amsterdam, Amsterdam, The Netherlands; 2 General and Pancreatic Surgery Department, Pancreas Institute, University and Hospital Trust of Verona, Verona, Italy; 3 Department of Surgery, Erasmus University Medical Center, Rotterdam, The Netherlands; 4 Department of Surgery, University Hospital Ghent, University of Ghent, Ghent, Belgium; 5 Department of Surgery, Radboud University Nijmegen Medical Centre, Nijmegen, The Netherlands; 6 Department of Surgery, Cancer Center Amsterdam, Amsterdam UMC, VU University, Amsterdam, The Netherlands; 7 Department of Surgery, Medisch Spectrum Twente, Enschede, The Netherlands; 8 The Pancreas and Liver Institute, Beth Israel Deaconess Medical Center and Harvard Medical School, Boston, MA USA; 9 Department of Surgery, Northshore University Health System, Chicago, IL USA

**Keywords:** Laparoscopy, Robot-assisted surgery, 3D, OSATS, Artificial organs

## Abstract

**Background:**

We tested the added value of 3D-vision on procedure time and surgical performance during robotic pancreatoduodenectomy anastomoses in biotissue. Robotic surgery has the advantage of articulating instruments and 3D-vision. Consensus is lacking on the added value of 3D-vision during laparoscopic surgery. Given the improved dexterity with robotic surgery, the added value of 3D-vision may be even less with robotic surgery.

**Methods:**

In this experimental randomized controlled cross-over trial, 20 surgeons and surgical residents from 5 countries performed robotic pancreaticojejunostomy and hepaticojejunostomy anastomoses in a biotissue organ model using the da Vinci® system and were randomized to start with either 3D- or 2D-vision. Primary endpoint was the time required to complete both anastomoses. Secondary endpoint was the objective structured assessment of technical skill (OSATS; range 12–60) rating; scored by two observers blinded to 3D/2D.

**Results:**

Robotic 3D-vision reduced the combined operative time from 78.1 to 57.3 min (24.6% reduction, *p* < 0.001; 20.8 min reduction, 95% confidence intervals 12.8–28.8 min). This reduction was consistent for both anastomoses and between surgeons and residents, *p* < 0.001. Robotic 3D-vision improved OSATS performance by 6.1 points (20.8% improvement, *p* = 0.003) compared to 2D (39.4 to 45.1 points, ± 5.5).

**Conclusion:**

3D-vision has a considerable added value during robotic pancreatoduodenectomy anastomoses in biotissue in both time reduction and improved surgical performance as compared to 2D-vision.

**Electronic supplementary material:**

The online version of this article (10.1007/s00464-020-07732-z) contains supplementary material, which is available to authorized users.

The morbidity rate of pancreatoduodenectomy is to a large extent caused by postoperative pancreatic fistula [1]. In recent years, minimally invasive PD has been introduced as a means to minimize the impact of surgery and thus enhance postoperative recovery [2]. Laparoscopic PD has the downside of non-articulating instruments and 2D-vision becomes especially apparent when creating pancreaticojejunostomy (PJ) and hepaticojejunostomy (HJ) anastomoses as during pancreatoduodenectomy [3, 4].

As stated by the 2020 international Miami evidence based guidelines on minimally invasive pancreatic resection, it is currently unclear whether the outcomes of general laparoscopic PD could benefit from 3D-vision [5]. More recently, the LAELAPS-3D2D study demonstrated a benefit in operative time and operative quality by 3D-laparoscopic reconstructive pancreatic surgery [4]. The use of an operating robot may facilitate minimally invasive PD as it overcomes some of the difficulties as encountered in regular laparoscopic surgery by improving dexterity and articulating instruments [6–8]. Given these benefits, one could argue that the added value of 3D-vision is therefore less in robotic surgery as compared to laparoscopic surgery. For upcoming robotic platforms, the question therefore remains whether robotic surgery should include 3D-vision [9, 10].

We aimed to determine the added value of 3D-vision, as compared to 2D-vision, when performing robotic pancreatoduodenectomy anastomoses in biotissue.

## Methods

### Study design

We conducted an experimental randomized controlled cross-over trial. The Consolidated Standards of Reporting Trials (CONSORT) guidelines where followed [11]. All participants were asked to complete a PJ and a HJ twice in an experimental setting using an artificial biotissue model simulating minimally invasive PD; once with 3D- and once with 2D-vision (see e-material 1 in the supplements for more detail on the procedures). The cross-over design with randomization was intended to minimize inter-observer differences and the impact of familiarity. The study was assessed and approved by the local ethics committee and performed in accordance with the Declaration of Helsinki [12]. The local ethics committee decided no informed consent was required for participating in this study, hereafter the study protocol was made available on the Dutch Trial Register: NL8063.

### Participants

Participants were invited from all 17 centers participating in the Dutch Pancreatic Cancer Group as well as from international collaborating centers. Sample size was calculated according to previous published data on laparoscopic 3D surgery, see statistical methods. The 20 participants included both expert surgeons and surgical residents, all of whom were capable of robotic suturing and completed a proficiency based robotic simulation curriculum [13]. Experience with minimally invasive PD was not required for participation. Stereoptic abilities, i.e*.,* 3D-vision capabilities, were assessed using a Randot Test (Stereo optical, Chicago, IL, USA). Reported side effects and preferences, and baseline demographics were collected using questionnaires (see eFigure 1 in Supplements). Participants (*n* = 0) were excluded if they had no 3D-vision abilities, > 200 s of arc [14].

### Intervention

The study was performed using the da Vinci® surgical robot in the operating rooms of 7 hospitals and at 2 experimental training facilities, always in the presence of the first author (MJZ) and performed during day-time hours. Participants first watched a 10 min instruction video and had an oral instruction before the start of the experiment.

**Fig. 1:**
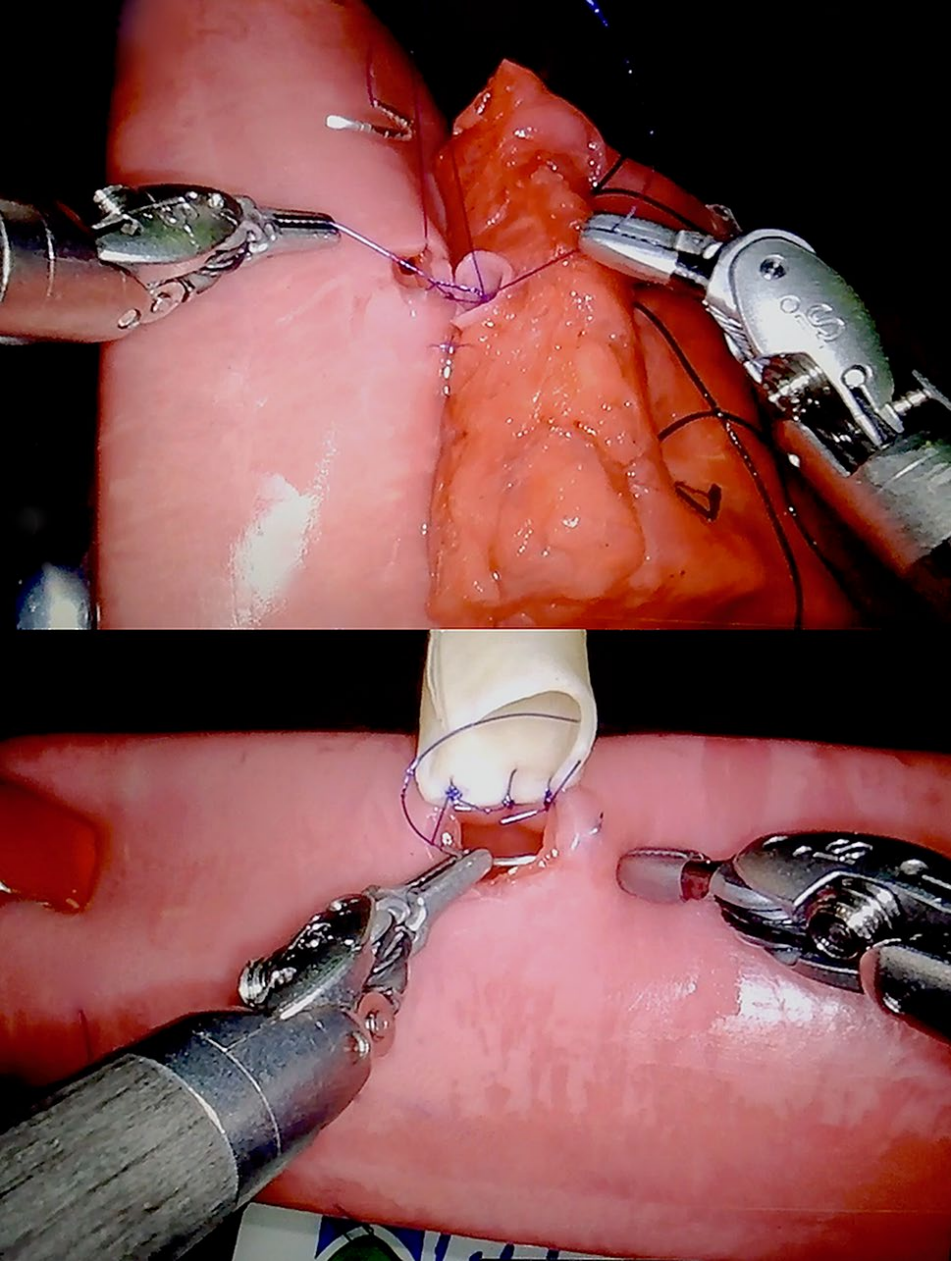
Robotic pancreatoduodenectomy anastomoses in biotissue set-up. Biotissue anastomoses set-up of the pancreaticojejunostomy (up) and hepaticojejunostomy (down)

A standardized patient setting was simulated using inanimate artificial, biotissue, organs (LifeLike BioTissue, Ontario, Canada) according to the Minimally Invasive Pancreatic Resection Organizing Committee method (See Fig. 1) [13]. The artificial organs included a long pancreas, long double layer small bowel, bile duct, vessel holder, and skin holder. Several minimally invasive training programs have incorporated these synthetic artificial organs, one of which shows face- and construct validity in training PD [13]. An integrated 3D HD da Vinci robotic laparoscope was used (Intuitive Surgical Inc., Sunnyvale California, USA), resolution (high-definition/1280 × 1024) and lighting conditions were identical between interventions. Participants used the identical set of robotic instruments and camera for all corresponding cross-over exercises. Ergonomic conditions were adjusted for all subjects before starting the experiment.

### Rating

All imaging material was presented to one rater and one validation rater anonymously, i.e., blinded for both the performing participant and for 3D/2D. The rater training was performed at University of Pittsburgh Medical Center. The validation rater was an experienced laparoscopic surgeon experienced in robotic pancreatoduodenectomy, who received a training in video rating. The validation rater received a random sample of 20 anastomoses of all anastomoses performed. The random sample was picked by a computer algorithm. Performance was rated using an objective structured assessment of technical skill (OSATS) validated by Birkmeyer et al. and Tam et al*.* (see Table 1) [13, 15, 16].

**Table 1:** Elements of objective structured assessment of technical skill (OSATS)

Grading definition	
1	Deficient/Traumatic
2	Lacking/Lacks finesse
3	Average
4	Skilled
5	Master/Flawless
Grading aspects and elucidation	
Gentleness Time and motion Instrument handling Flow of operation Tissue exposure Summary	Gentle tissue handling that does not result in injury Economy of motion, maximum efficiency Fluid use of instruments without awkwardness Smooth transitions from one part of the operation to another Retraction that allows for good visualization and proper tissue alignment Overall assessment of technical skill

5-Point rating scale modified for static surgical environments [15, 16]

### Randomization

Randomization was done with SPSS (SPSS Inc., Chicago, IL, USA) by the study coordinator. Participant data were anonymized by using a 4-digit code, and the principal investigator and study coordinators were the only ones with access to the decoding document.

### Outcomes

The primary outcome was the difference in total operative time expressed in minutes and percentages. Secondary outcome was surgical performance according to the OSATS score (attainable range 12–60). Other outcomes were the difference in operative time for the PJ and HJ; participant’s preference for 3D or 2D, and side effects of 3D-vision.

### Statistical methods

The sample size estimation for this trial was based on a previous randomized study assessing 3D laparoscopy (LAELAPS-3D2D) [4]. Assuming a pooled standard deviation of 10 min, the study would require a sample size of: 10 participants for each group, i.e., a total sample size of 20 participants to achieve a power of 90% and a level of significance of 5% (two sided), for detecting a true difference in means between the test and the reference group of 15 min. Power calculation did not focus on participant categorization as surgeons or residents since no statistical difference was found in the LAELAPS-3D2D study, nonetheless, we reported on these results for our primary and secondary outcome as means of sensitivity analysis [4].

Data were analyzed using IBM SPSS statistics for Windows version 24 (IBM Corp, Armonk, NY, USA). Normally distributed continuous data were presented as means and standard deviations (±). Non-normally distributed continuous data were presented as medians and interquartile ranges (IQRs) or 95% confidence intervals (95% CI). Categorical (binary, nominal, and ordinal) data were presented as frequencies and percentages. Likert-Scale ordinal data were also presented in means and standard deviations, as this allows more insight into the effect size [17]. A two-tailed *p* value of less than 0.05 was considered statistically significant. Missing data were corrected by excluding the corresponding missing part of the video of both the intervention and control procedure into the analysis (*n* = 1, HJ, 2 min due to recording failure). Differences in anastomosis times were analyzed paired-wise according to the performing participant.

Baseline demographics were compared with Student’s t-test for normally distributed data, Chi-squared test for frequencies in one or more categories, and Mann–Whitney *U* test for non-normally distributed data. Primary outcomes were analyzed using the Wilcoxon signed-rank test, since the comparison was paired-wise. Secondary outcomes were analyzed using Wilcoxon signed-rank test and analysis was performed with a Student’s t-test after normality was assessed.

## Results

### Participants

**Fig. 2:**
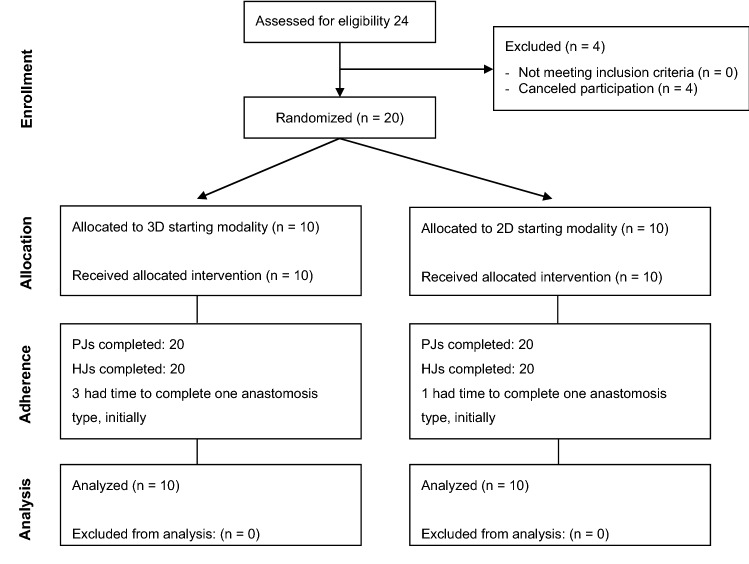
Flowchart of inclusion. *PJ* Pancreaticojejunostomy, *HJ* hepaticojejunostomy

In the period December 2017 to January 2019, over 30 surgeons and surgical residents were invited, resulting in 20 participants of which 16 participants completed the experiment in a single session. For detailed information on the numbers of participants randomly assigned, their performed procedures, and data analyzed, see Fig. 2. The 20 participants completed 40 PJs and 40 HJs.

### Baseline demographics

Surgeons (*n* = 14) and residents (*n* = 6) from 5 countries (Belgium, Estonia, Italy, Japan, the Netherlands) participated. The two groups, i.e*.,* start with 3D or 2D, were comparable for baseline characteristics, robotic experience (13/14 surgeons and 1/6 residents had performed robotic surgery in clinical practice as console surgeon), hand dominance, vision correction and 3D-vision abilities. Mean age was 35 years (± 8) and 16/20 participants were male (see Table 2). Among those who had performed robotic surgery, the median robotic experience was 1 year (IQR 1–2) with a median annual volume of 20 advanced robotic procedures (IQR 1–40). Of all participants, 8 (40%) performed MIPD, of which the median number of MIPDs performed was 23 (IQR 13–48).

**Table 2:** Participant characteristics

	Total (*N* = 20)	3D-first (*N* = 10)		2D-first (*N* = 10)	*p* value
Age (years)	35 ± 8	36 ± 9		36 ± 4	0.719^a^
Male (%)	16 (80)	8 (80)		8 (80)	1.000^b^
Experience in minimally invasive surgery					
Surgeons	14 (80)	7 (80)		7 (80)	1.000^b^
Clinical robotic experience^d^	13/14	6/7		7/7	
Residents	6 (30)	3 (30)		3 (30)	1.000^b^
Clinical robotic experience^d^	1/6	0/3		1/3	
Years of robotic experience	1 (0–1.8)	1 (0–1.3)		1 (0–2.0)	0.872
Number of advanced robotic procedures performed annually	20 (1–40)	10 (0–36)		30 (4–50)	0.657^c^
Performed minimally invasive pancreatoduodenectomies (MIPD) (%)	8 (40)	4 (40)		4 (40)	1.000^b^
Number of MIPDs performed	23 (13–48)	16 (10–43)		33 (21–48)	0.343^c^
Hand dominance					0.589^b^ (*X*_2_ = 1.059)
Right (%)	18 (85)	8 (80)	9 (90)		
Left (%)	2 (10)	1 (10)	1 (10)		
Ambidextrous (%)	1 (5)	1 (10)	0 (-)		
Vision correction (%)	8 (40)	5 (50)	3 (30)		0.581^b^
Minimal degrees of stereopsis, seconds of arc	50 (20–130)	60 (40–80)	100 (60–200)		0.108^c^

Values are mean ± SD, median (quartile 1 to quartile 3) or *n* (percentage)

^a^Students *t*-test

^b^Chi-square test

^c^Mann–Whitney *U* test

^d^As console surgeon

### Primary outcomes

**Fig. 3:**
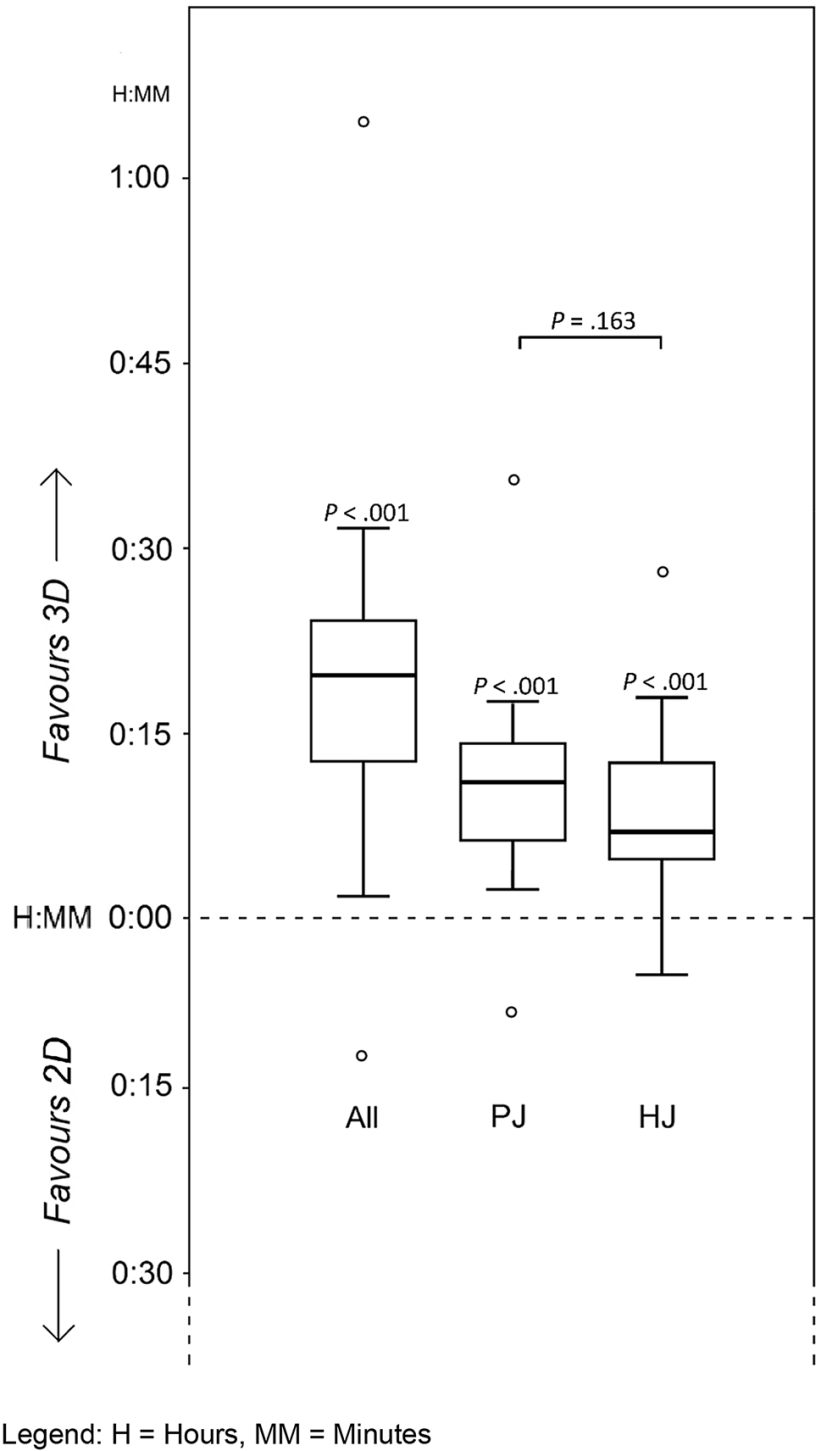
Time reduction to complete both anastomoses. *H* Hours, *MM* minutes

The mean operative time to complete both anastomoses was 57.3 (± 14.3) min (median 56.0, IQR 47.5–67.3) with 3D-vision and 78.1 (± 20.5) min (median 75.5, IQR 69.3–95.5) with 2D-vision. The median reduction in total operative time with 3D was 20.8 min, 95%CI 12.8–28.8, (*p* < 0.001). The relative reduction in operative time with 3D was 24.6% (± 17.3), 95% CI 15.6–33.5 (*p* < 0.001), see Fig. 3.

The median operative time for the PJ was 37.2 min (IQR 30.5–43.5) in 3D and 49.2 min (IQR 44.3–58.0) in 2D. The median reduction in PJ operative time with 3D was 11.2 min, 95% CI 6.1–15.5 (*p* < 0.001), or 25.2%. The median operative time for the HJ was 19.5 min (IQR 16.4–27.2) in 3D and 27.5 min (IQR 23.5–40.1) in 2D. The median reduction in HJ operative time in 3D was 7.2 min, 95% CI 4.4–13.2 (*p* < 0.001), or 29.6%. The benefit of 3D did not differ between both anastomoses (*p* = 0.163) (see Fig. 3). The relative improvements in operative time were 25.2%, 95% CI 12.1–30.1 (*p* < 0.001), for PJ, and 29.6%, 95% CI 14.5–40.4 (*p* < 0.001), for HJ.

### Secondary outcomes

The mean OSATS for both anastomoses was 45.1 (± 6.2) points using 3D-vision and 39.4 (± 9.2) points using 2D-vision, with attainable scores between 12 and 60. The mean overall improvement in the OSATS for both anastomoses with 3D-vision was 6.1 points, ± 5.5, *p* = 0.003. The relative improvement was 20.8%.

Of all participants, 20/20 participants stated to prefer 3D-vision, whereas 4/20 reported side effects. Minor side effects, e.g., minor eyestrain, were reported by 3/20 participants and 1/20 participants reported serious side effects, e.g., serious headache in relation to 3D-vision. Surprisingly, 2 participant reported side effects in relation to 2D-vision (see eTable 2 in Supplements).

The validation grading mean OSATS for both anastomoses was 39.0 (± 4.3) points using 3D-vision and 31.7 (± 1.9) points using 2D-vision with a mean overall improvement in the OSATS for both anastomoses with 3D-vision of 7.3 points, ± 3.2, *p* = 0.027.

### Surgeons and residents

The median reduction in operative time with 3D-vision was 20.0 min, 95% CI 14.6–33.1 (*p* = 0.002), or 29.8%, for surgeons and 21.0 min, 95% CI 8.2–35.0 (*p* = 0.138), or 19.4%, for residents. The reduction in operative time with 3D did not differ significantly between surgeons and residents (*p* = 0.279).

The mean overall improvement in the OSATS for both anastomoses with 3D-vision was 6.0 points, ± 5.1, *p* = 0.005, or 17.9%, for surgeons and 6.4 points, ± 6.7, *p* = 0.099, or 20.8%, for residents. The improvement did not differ significantly between surgeons and residents, *p* = 0.615.

When stratifying outcome of surgeons in the 50% with the most experience and the 50% with the least experience we found a similar benefit of 3D-vision compared to 2D-vision. For the stratum with higher experience median 52.5 min versus 73.50 min, *p* = 0.012, respectively. For the stratum with lower experience median 58.5 min versus 78.0 min, *p* = 0.025, respectively. For the stratum with higher experience mean OSATS of 47.6 versus 42.3, *p* = 0.26, respectively*.* For the stratum with lower experience median OSATS of 42.0 versus 36.5, *p* = 0.021, respectively*.*

## Discussion

This first experimental randomized study to examine the impact of robotic 3D-vision during pancreatoduodenectomy anastomoses in biotissue found that 3D-vision reduced operative time by 25% and improved surgical performance with 21% (based on OSATS), as compared to 2D-vision. The benefit of 3D-vision on operative time, and its effect size, was similar between surgeons and residents during robotic surgery in an experimental setting, similar between surgeons with more and less experience, and was present for both the pancreatic and bile duct anastomoses.

It seems that 3D-vision had a similar, or even somewhat higher, advantage for surgical time and surgical performance with robotic surgery as compared to laparoscopic surgery. The present study followed the LAELAPS-3D2D randomized trial that studied the same outcomes for 3D laparoscopy [4]. In that study, 3D laparoscopy improved surgical time with 20% and surgical performance with 12%. A recent systematic review confirmed that 3D laparoscopy improved operative time and reduced the number of performance errors when compared to 2D-laparoscopy in abdominal surgery and simulation surgery [3]. Recent randomized trials on laparoscopic versus open pancreatoduodenectomy did not take 3D-vision into account [18–20].

Birkmeyer et al*.* established that the complication rates nearly halves for every point increase in the numerical rating [16]. Lower OSATS performance scores have also been shown predictive of postoperative pancreatic fistulas in robot-assisted pancreatoduodenectomy [21, 22]. In this study, 3D-vision improved both the surgical performance and operative time. This is especially important considering the implementation of new robotic platforms and articulating laparoscopic instruments [9]. Clinical studies will have to confirm these suggestions.

What are disadvantages of 3D-vision? As confirmed in this study, some surgeons report discomfort when using 3D-vision. These side effects are mostly minor and appear less than previously reported for 3D laparoscopy potentially due to the more stable camera position with robotic surgery. Then again, in this study all participants had at least average 3D-vision capabilities, explaining the limited amount of complaints. People who are unable to see stereoptically experience adverse effects of nausea and eye strain [23]. This study used the da Vinci® system. Currently, several robotic systems are in development and it is currently unknown whether these will include 3D-vision [9].

This study has some limitations. First, this was a randomized trial in an experimental and not in a clinical setting. A clinical study comparing the benefits of 3D-vision would, however, seem unlikely especially in robotic surgery where 3D-vision is standard of care. This study is rather theoretical since 3D-vision is standard for current robotic surgery. The reported observations could also be translated to conventional laparoscopy which does not include 3D-vision routinely and to new robotic systems that aim to improve viewing conditions in minimally invasive surgery. A bias in familiarity with robotic surgery combined with 3D-vision seems unlikely, as there was no significant difference between surgeons and residents, and sensitivity analysis revealed that the benefit of 3D-vision was similar when stratifying the groups for experience. Second, participants were recruited on an invitational basis. This could have caused a selection bias toward participants with better skills or better stereoptic capabilities (mean seconds of arc of stereopsis of 50 (IQR 20–130)) [14]. Third, blinding the participants for the intervention was clearly not possible. However, since the video raters were blinded for 2D and 3D-visions as they viewed all videos in 2D only, observer bias will not have played a relevant role. Unfortunately, the subjective nature of rating cannot be avoided, as an automated evaluation is not established and could also be prone to methodological problems.

Strengths of the current study include the randomized design, the use of international participants with varying expertise, observers blinded for 3D or 2D regarding OSATS outcomes, and standard description of stereoptic capabilities. With the use of artificial organs, confounders of patient variables are eliminated, e.g., variations in pancreas morphology.

In conclusion, 3D-vision has a substantial, probably larger than expected, contribution to the value of robotic surgery, as compared to 2D-vision, with 25% reduction in operative time of completing both robotic biotissue PJ and HJ and 21% improved surgical performance. New robotic platforms and articulating laparoscopic instruments are advised to include 3D-vision.

## Electronic supplementary material

Below is the link to the electronic supplementary material.

Supplementary file 1 (DOCX 356 kb)
