# Supplementary material for: Added value of 3D-vision during robotic pancreatoduodenectomy anastomoses in biotissue (LAEBOT 3D2D): a randomized controlled cross-over trial
Source: Surg Endosc. 2020 Jul 13;35(6):2928–35. doi: 10.1007/s00464-020-07732-z (PMC8116254; doi:10.1007/s00464-020-07732-z)
Supplement: Supplementary file 1 — (DOCX 356 kb) [file 464_2020_7732_MOESM1_ESM.docx]

# SUPPLEMENTAL DIGITAL CONTENT

## List of supplemental digital content

SDC 1. Text and figure that illustrate the detailed procedures.

SDC 2. Questionnaire on participant baseline demographics.

SDC 3. Online-Only Supplementary Table 2.

## SDC 1. Text and figure that illustrate the detailed procedures.

Caption: Text and figure that illustrate the detailed procedures. Adapted from Zwart et al., 2019, HPB [4].

### Hepaticojejunostomy

**One hepatojejunostomy is created using the following procedure comprising eight standing sutures.**


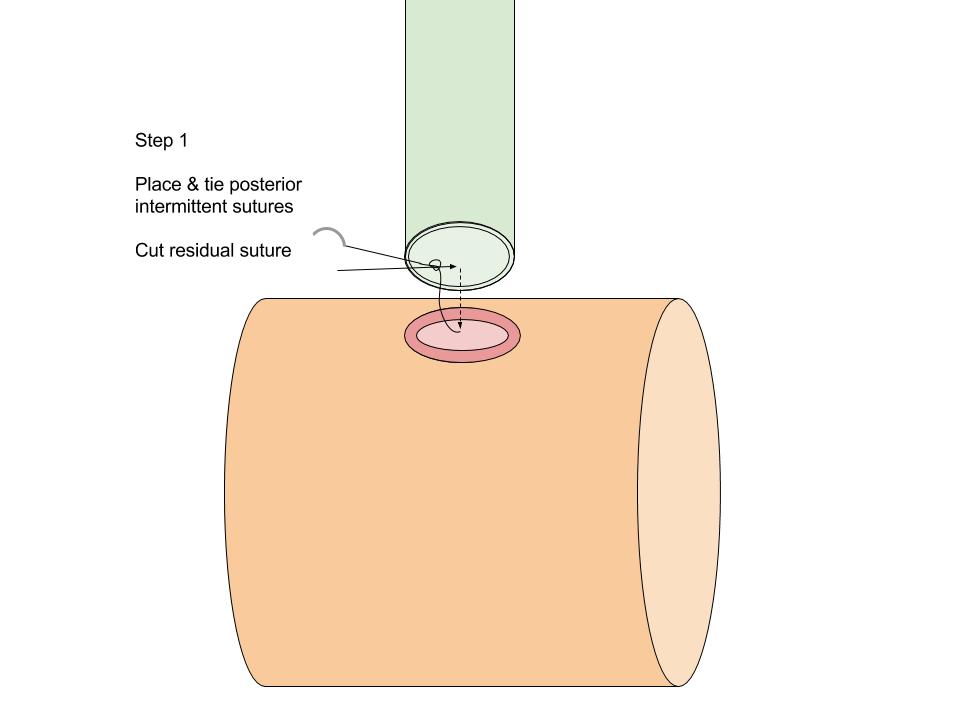
**Step 1. At the 6 O’clock position, a** suture 5-0 14CM 17MM RB-1 Needle is driven from inside the bile duct lumen through the outside of the jejunum. A double knot is created followed by 3 loop knots. Scissors are used to cut of the suture at the resulting standing knot. The leftover material is used in the standing created at step 2.

**Step 2. At the half past 4 position, the leftover** suture 5-0 14CM 17MM RB-1 Needle from step 1 is driven from inside the bile duct lumen through the outside of the jejunum. A double knot is created followed by 3 loop knots. Scissors are used to cut of the suture at the resulting standing knot. The needle is placed on the right-hand corner of the set-up and extruded with the help of the assistant.


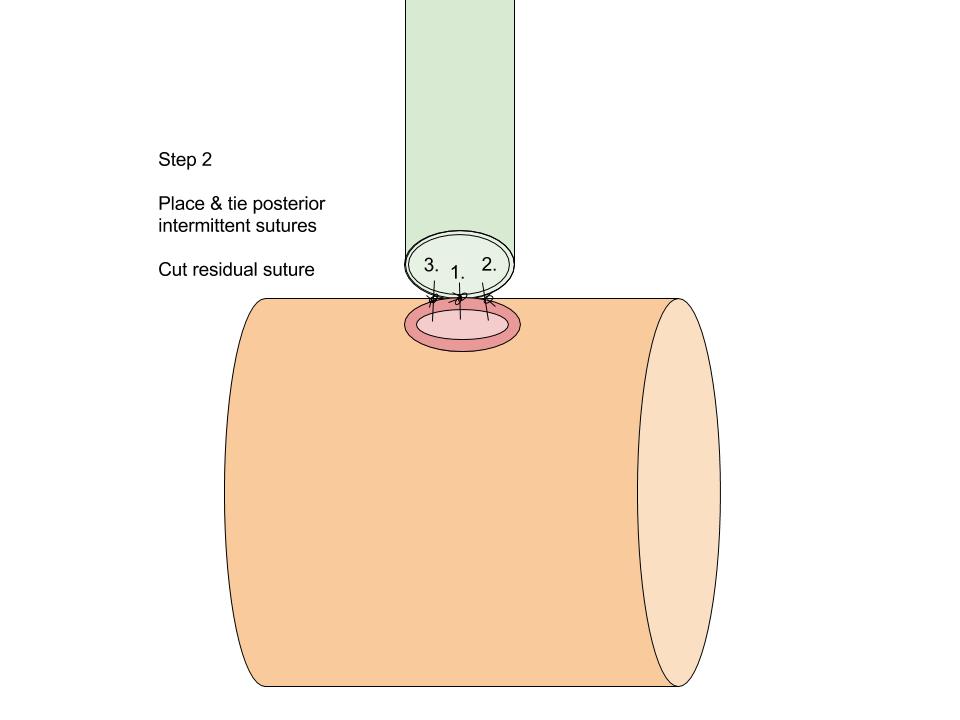
**Step 3. At the half past 7 position, a** suture 5-0 14CM 17MM RB-1 Needle is driven from inside the bile duct lumen through the outside of the jejunum. A double knot is created followed by 3 loop knots. Scissors are used to cut of the suture at the resulting standing knot. The leftover material is used in the standing knot created at step 4.

**Step 4. At the 3 O’clock position, the leftover** suture 5-0 14CM 17MM RB-1 Needle from step 3 is driven from outside of the bile duct lumen through the inside of the jejunum. A double knot is created followed by 3 loop knots. Scissors are used to cut of the suture at the resulting standing knot. The needle is placed on the right-hand corner of the set-up and extruded with the help of the assistant.


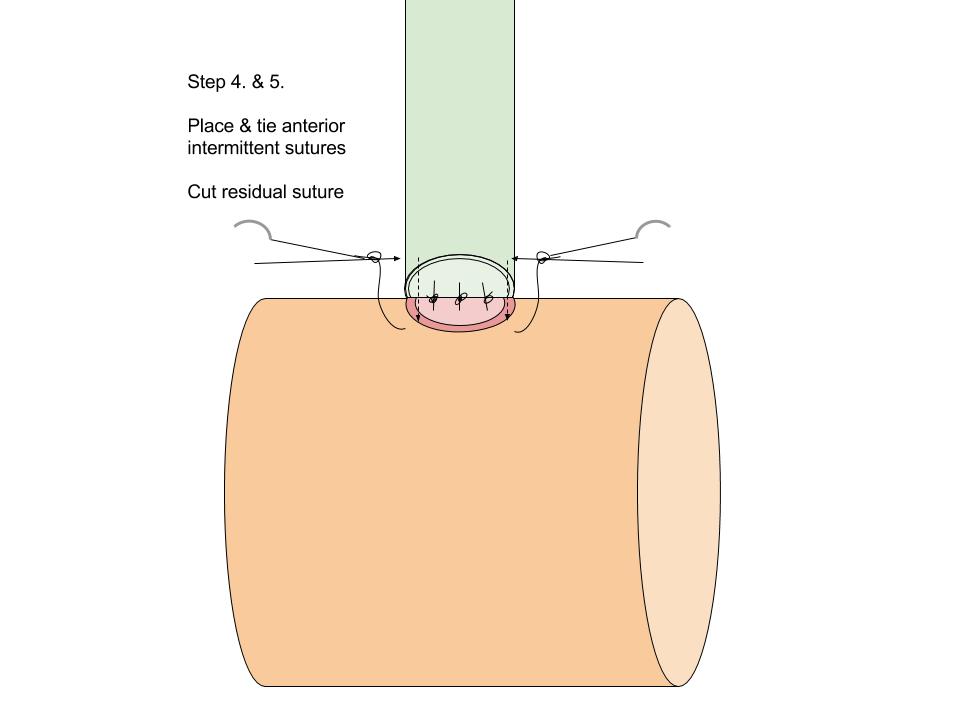
**Step 5. At the 9 O’clock position, a** suture 5-0 14CM 17MM RB-1 Needle is driven from outside the bile duct through the inside of the jejunum. A double knot is created followed by 3 loop knots. Scissors are used to cut of the suture at the resulting standing knot. The leftover material is used in the standing knot created at step 6.

**Step 6. At the half past 1 position, a** suture 5-0 14CM 17MM RB-1 Needle from step 5 is driven from outside the bile duct lumen through the outside of the jejunum. A double knot is created followed by 3 loop knots. Scissors are used to cut of the suture at the resulting standing knot. The needle is placed on the right-hand corner of the set-up and extruded with the help of the assistant.


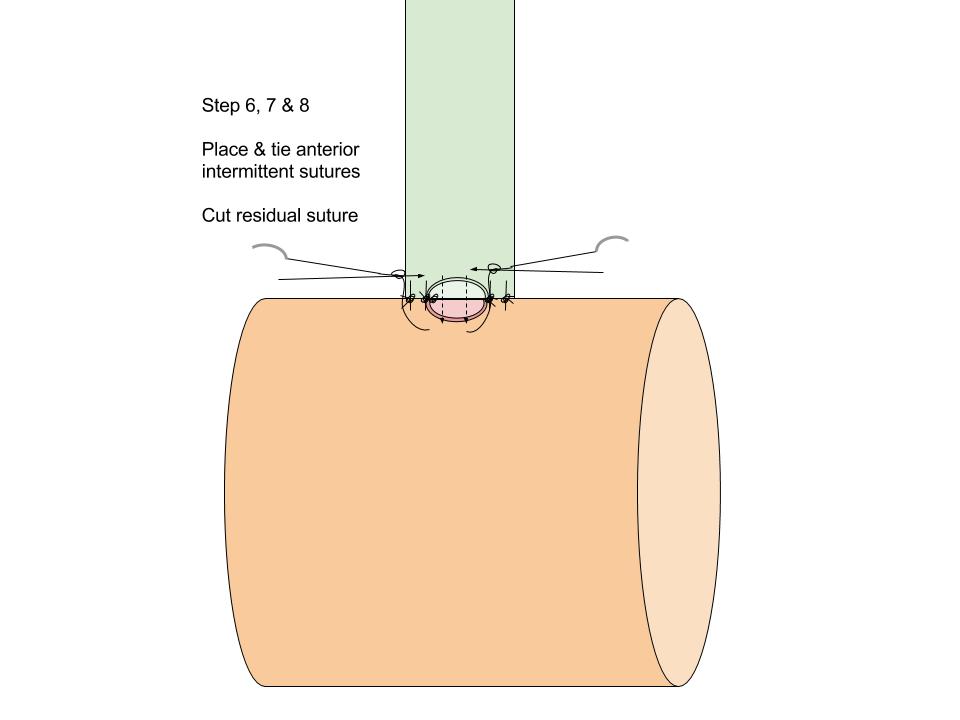
**Step 7. At the half past 10 position, the leftover** suture 5-0 14CM 17MM RB-1 Needle is driven from outside of the bile duct lumen through the inside of the jejunum. A double knot is created followed by 3 loop knots. Scissors are used to cut of the suture at the resulting standing knot. The leftover material is used in the standing knot created at step 8

**Step 8. At the 12 O’clock position, a** suture 5-0 14CM 17MM RB-1 Needle from step 7 is driven from outside the bile duct lumen through the outside of the jejunum. A double knot is created followed by 3 loop knots. Scissors are used to cut of the suture at the resulting standing knot. The needle is placed on the right-hand corner of the set-up and extruded with the help of the assistant.

### Pancreaticojejunostomy

**One pancreaticojejunostomy is created using the following modified Blumgart method [24].**

**Step 1.**


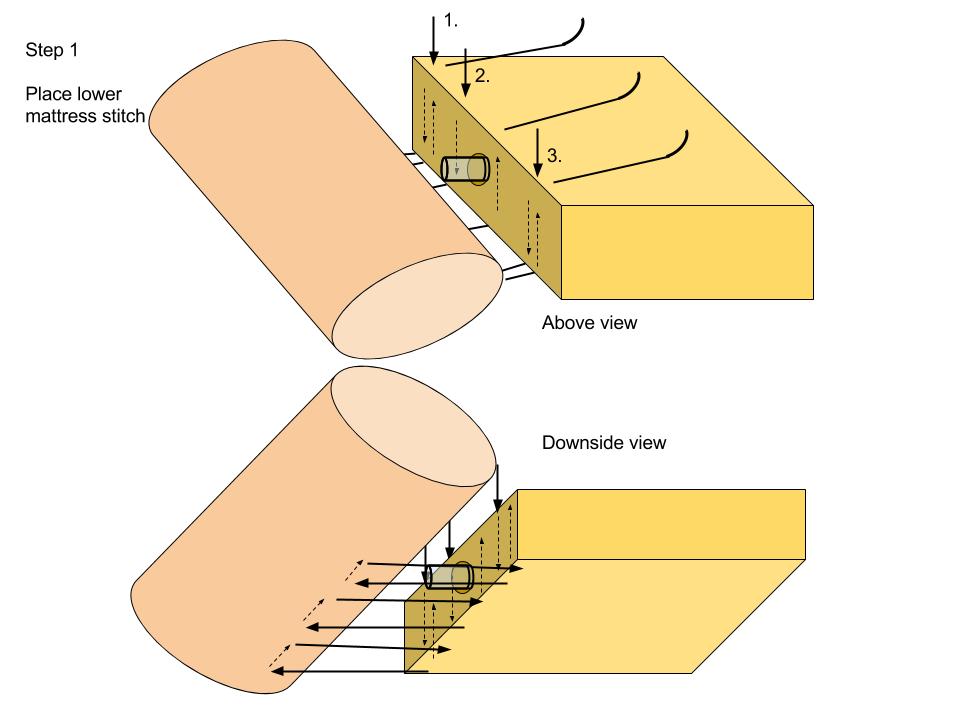
**Step 1.a. At the cranial position of the pancreas, denoted with 1., a** suture 2-0 23CM 36MM CT-1 needle is driven through the anterior plane of the pancreas to the posterior plane. The needle is then driven through the jejunum in a cranial to caudal direction and back through the pancreas from posterior to anterior to create a mattress stitch. The suture is tightened, a double knot is tied and followed by two loops. The needle is then placed out of the working field.

**Step 1.b. At the central position of the pancreas, just cranial of the pancreatic duct, denoted with 2., a** suture 2-0 23CM 36MM CT-1 needle is driven through the anterior plane of the pancreas to the posterior plane. The needle is then driven through the jejunum in a cranial to caudal direction and back through the pancreas, just caudal from the pancreatic duct, from posterior to anterior to create a mattress stitch. A stent is inserted in the pancreatic duct. The suture is tightened, a double knot is tied and followed by three loops. The needle is then placed out of the working field.

**Step 1.c. At the caudal position of the pancreas, denoted with 3., a** suture 2-0 23CM 36MM CT-1 needle is driven through the anterior plane of the pancreas to the posterior plane. The needle is then driven through the jejunum in a cranial to caudal direction and back through the pancreas from posterior to anterior to create a mattress stitch. The suture is tightened, a double knot is tied and followed by three loops. The needle is then placed out of the working field.

**Step 2.**


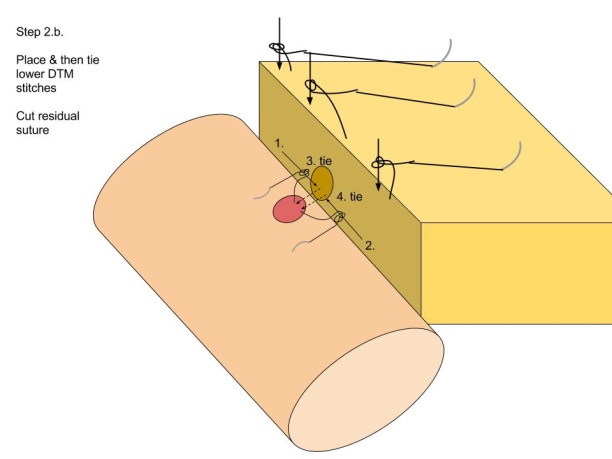
**Step 2.a.** The ductal stent is removed from the pancreatic duct and extracted by the assistant.

**Step 2.b. At the ‘legs of the star’, t**he first posterior duct-to-mucosa stitches are created **with a** 5-0 14CM 17MM RB-1 Needle from inside the pancreatic duct lumen through the outside of the jejunum. A double knot is created followed by 3 loop knots. Scissors are used to cut of the suture at the resulting standing knot. The leftover material is used in the succeeding standing stitch created or the needle is placed on the right-hand corner of the set-up and extruded with the help of the assistant.


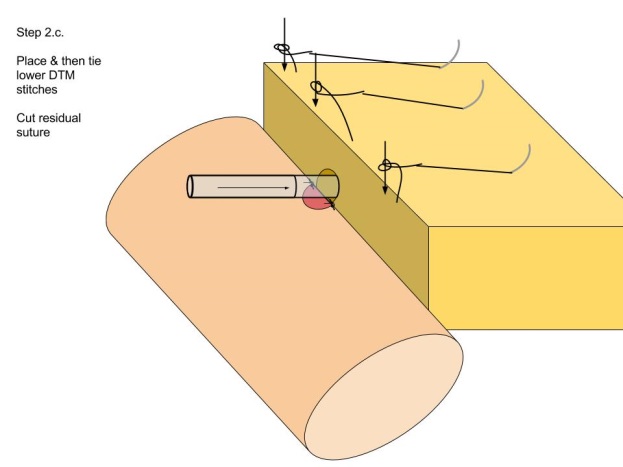
**Step 2.c.** The stent is handed by the assistant. The ductal stent is inserted in the pancreatic duct and in the jejunum.

**Step 2.d.** The first and second anterior duct-to-mucosa stitches are created **at the ‘arms of a star’ position, a** suture 5-0 <14CM 17MM RB-1 Needle is driven from outside of the jejunum through the inside of the pancreatic duct lumen. A double knot is created followed by 3 loop knots. Scissors are used to cut of the suture at the resulting standing knot. The leftover material is used in the succeeding standing stitch created.

**At the ‘head of the star’ position, the leftover** suture 5-0 <14CM 17MM RB-1 Needle from step 2.d. is driven from outside of the jejunum through the inside pancreatic duct. A double knot is created followed by 3 loop knots. Scissors are used to cut of the suture at the resulting standing knot. The needle is placed on the right-hand corner of the set-up and extruded with the help of the assistant.

**Step 3.**


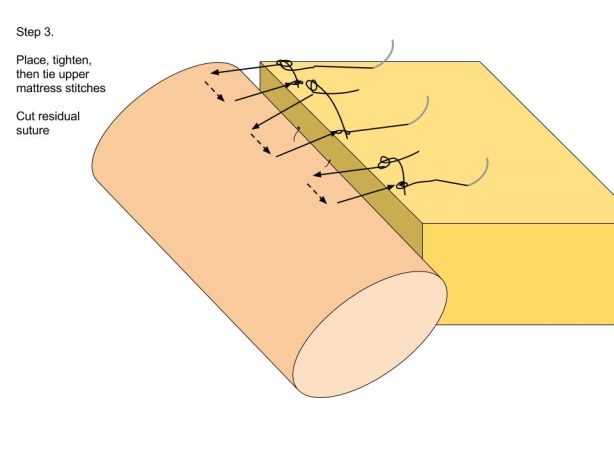
**Step 3.a. At the cranial position of the pancreas, denoted with 1., the** suture 2-0 23CM 36MM CT-1 needle is driven through the jejunum in a cranial to caudal direction, just laterally from the knot created at step 1.a.. The suture is tightened, a double knot is tied and followed by three loops. Scissors are used to cut of the suture at the resulting knot and the leftover material is placed outside of the working field or handed directly to the assistant.

**Step 3.b. At the central position of the pancreas, denoted with 2., the** suture 2-0 23CM 36MM CT-1 needle is driven through the jejunum in a cranial to caudal direction, just laterally from the knot created at step 2.a.. The suture is tightened, a double knot is tied and followed by three loops. Scissors are used to cut of the suture at the resulting knot and the leftover material is placed outside of the working field or handed directly to the assistant.

**Step 3.c. At the caudal position of the pancreas, denoted with 3., the** suture 2-0 23CM 36MM CT-1 needle is driven through the jejunum in a cranial to caudal direction, just laterally from the knot created at step 3.a.. The suture is tightened, a double knot is tied and followed by three loops. Scissors are used to cut of the suture at the resulting knot and the leftover material is placed outside of the working field or handed directly to the assistant.

## SDC 2. Questionnaire on participant baseline demographics

Caption: Questionnaire on participant baseline demographics


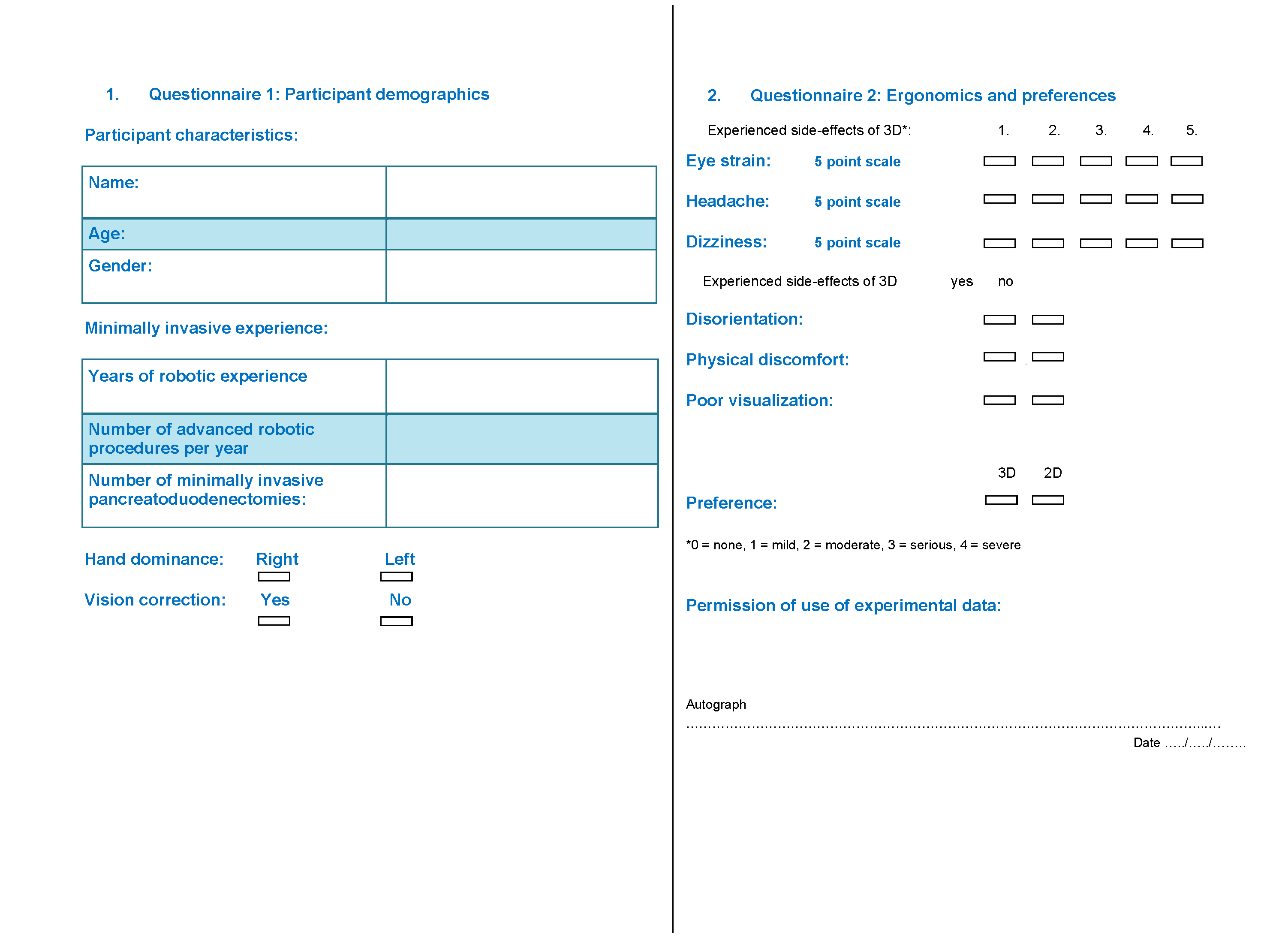
Legend: -

## SDC 3. Complaints caused by 3D-vision

Caption: Complaints caused by 3D-vision

Legend: Values in parentheses are percentages. 2 participant reported side effects in relation to 2D-vision of moderate eye strain and moderate headache.

| **Completed survey**  ***N* = 20** | **None** | | **Minor** | | **Moderate** | | **Serious** | **Severe** |
| --- | --- | --- | --- | --- | --- | --- | --- | --- |
| **Eye strain** | **18 (90%)** | | **2 (10%)** | | **0 (0%)** | | **0 (0%)** | **0 (0%)** |
| **Headache** | **18 (90%)** | | **1 (5%)** | | **0 (0%)** | | **1 (5%)** | **0 (0%)** |
| **Dizziness** | **17 (75%)** | | **2 (10%)** | | **0 (0%)** | | **1 (5%)** | **0 (0%)** |
|  | | **Yes** | | **No** | |  | | |
| **Disorientation** | | **0 (0%)** | | **20 (100%)** | |  | | |
| **Physical discomfort** | | **1 (5%)** | | **19 (95%)** | |  | | |
| **Poor visualization** | | **0 (0%)** | | **20 (100%)** | |  | | |
| **Preferred 3D** | | **20 (100%)** | | **0 (0%)** | |  | | |
